# An attention-enhanced hybrid CNN-BiLSTM framework for multiclass epileptic seizure recognition using EEG signals

**DOI:** 10.3389/fncom.2026.1949040

**Published:** 2026-10-06

**Authors:** Bobbinpreet Kaur, Sheenam Middha, Aleem Ali, Noman Khan, Reham Alhejaili, Ahmed H. Almutairi

**Affiliations:** 1 Department of Computer Science and Engineering, University Institute of Engineering (UIE) Chandigarh University, Gharuan, Punjab, India; 2 Department of Computer Science and Engineering, Sejong University, Seoul, Republic of Korea; 3 Department of Computer Science and Artificial Intelligence, College of Computer Science and Engineering, University of Jeddah, Jeddah, Saudi Arabia; 4 Department of Computer Science, Applied College, Northern Border University, Arar, Saudi Arabia

**Keywords:** anesthesia monitoring, BiLSTM, CNN, consciousness states, deep learning, EEG classification, epilepsy, seizure detection

## Abstract

**Introduction:**

Electroencephalogram (EEG) signal classification plays a pivotal role in brain-computer interfaces (BCIs) and the diagnosis of neurological disorders. However, the non-stationary, non-linear, and low signal-to-noise ratio characteristics of EEG signals make accurate feature extraction challenging. Conventional deep learning approaches often struggle to simultaneously capture localized transient patterns, deep temporal dependencies, and long-range contextual relationships within EEG signals.

**Methods:**

To address these challenges, an optimized end-to-end hybrid deep learning framework, termed CNN–BiLSTM–MHSA, is proposed for multiclass epileptic seizure recognition. The architecture sequentially integrates a multilayer one-dimensional Convolutional Neural Network (CNN) for localized feature extraction, a Bidirectional Long Short-Term Memory (BiLSTM) network for capturing forward and backward temporal dependencies, and a Multi-Head Self-Attention (MHSA) mechanism for dynamically emphasizing discriminative neurological events. Robust preprocessing includes Butterworth bandpass filtering, Z-score normalization, and data augmentation using Gaussian noise injection and amplitude scaling. The framework was evaluated on the UCI/Kaggle Epileptic Seizure Recognition dataset comprising 11,500 EEG segments distributed across five classes.

**Results:**

The proposed framework achieved an overall accuracy of 98.83%, precision of 98.95%, recall (sensitivity) of 98.84%, F1-score of 98.90%, specificity of 99.71%, balanced accuracy of 98.84%, ROC-AUC of 99.80%, and PR-AUC of 99.72%. Class-wise accuracies exceeded 99% for most categories, with one seizure class achieving perfect recognition. Comparative evaluation further demonstrated that the proposed framework outperformed several recent state-of-the-art EEG seizure classification methods.

**Discussion:**

The superior performance can be attributed to the complementary representation capabilities of the hybrid architecture. CNN layers effectively learn localized signal patterns, BiLSTM captures bidirectional temporal dependencies, and MHSA enhances the representation of long-range contextual relationships by assigning greater importance to informative temporal regions. The combination of robust preprocessing and data augmentation further contributes to improved generalization and classification reliability.

## 1 Introduction

EEG is a non-invasive neuroimaging technique that measures the brain's high temporal resolution electric activity. In the clinical environment, EEG signals are essential in anesthetic monitoring, epilepsy diagnosis and assessing altered states of consciousness (Raghu et al., 2020). Manual interpretation of EEG by trained neurologist is subject to fatigue-induced error and subjective, time consuming. In this regard, automated EEG analysis based on machine learning and deep learning has gained a lot of research interest. One of the most important aspects to patient safety is the ability to detect seizures accurately and timely. Likewise, accurate monitoring of anesthesia status prevents awareness during anesthesia, which is linked with post-traumatic stress disorder and patient outcome (Martins et al., 2026). The monitoring of depth of anesthesia by EEG has thus become a major part of contemporary perioperative management. Although these developments are significant, automated EEG signal analysis is still a daunting task. EEG signals are characterized by low amplitude (typically 10–100 microvolts), sensitivity to artifacts (eye movements, muscle activity, electrode noise), high inter-subject and intra-session variability and complex non-stationary temporal dynamics that span multiple frequency bands. The traditional feature engineering methods, which involve manually designed features like power spectral density, wavelet coefficients, or common spatial patterns, are time-consuming, task-specific, and may not fully account for the complexity of EEG spatiotemporal data. With the emergence of deep learning, a paradigm shift has taken place in the processing of EEG signals. Convolutional Neural Networks are known to be good at extracting local spatial and spectral features with learned filter banks, and Recurrent Neural Networks (RNNs), specifically Long Short-Term Memory (LSTM) networks, are capable of capturing the sequential temporal dependencies that are present in EEGs time-series. Recently, transformer-based attention mechanisms have been shown to have better performance in selectively attending to the most relevant temporal features for a given task, overcoming the inability of RNNs to attend to different parts of their context window. The conventional EEG classification methods like support vector machine (SVM) and linear discriminant analysis (LDA) rely on hand-crafted feature engineering, but they cannot reflect the non-linear, non-stationary dynamics of EEG signals (Arshath and Karthikeyan, 2026). In contrast to deep learning architectures, these are architectures that rely on a human-designed hierarchy to extract features directly from raw signals. CNNs are good at learning local temporal structure, while LSTM networks are good at learning long-range temporal structure. However, there are drawbacks to architectures that do not rely on connection to one another: CNNs have no notion of temporal context, and recurrent networks are inefficient in processing long sequences. Hybrid models of CNNs and recurrent layers, have shown better results on EEG classification tasks (Lashkari et al., 2026). Additionally, the addition of attention mechanisms allows models to attend to clinically relevant temporal regions, leading to greater classification accuracy and interpretability (Zhai et al., 2026). Although these progress have been made, existing work has not yet explored a comprehensive framework that combines advanced preprocessing, CNN feature extraction, Bidirectional LSTM temporal modeling, multi-head self-attention, and cross-classification with extensive cross-validation schemes for multi-class EEG consciousness classification (Tiwari et al., 2026).

The novelty of this study lies in the development of a unified CNN–BiLSTM–Multi-Head Self-Attention framework that jointly models local EEG characteristics, bidirectional temporal dependencies, and long-range contextual relationships. Unlike conventional CNN-based approaches that primarily learn local signal representations, the proposed framework incorporates BiLSTM to capture temporal dependencies in both forward and backward directions and subsequently employs MHSA to selectively model globally relevant temporal information.

This paper presents such a framework. The key contributions of this work are:

- A novel hybrid CNN-BiLSTM-attention architecture tailored for multi-class EEG consciousness state classification.
- A complete EEG preprocessing pipeline that includes bandpass filtering, notch filtering, *Z*-score normalization and fast Fourier transform (FFT)-based spectral feature fusion.
- Data augmentation technique for improving model generalization by injecting gaussian noise into the data.
- Extensive evaluation using 5-fold stratified cross-validation with metrics including accuracy, precision, recall, F1-score, and specificity.
- Visualization of self-attention weights to provide interpretable temporal salience maps highlighting EEG regions that contribute to model predictions.
- State-of-the-art performance surpassing existing benchmark methods on the Epileptic Seizure Recognition dataset.

The rest of this paper is organized as follows. The related work is summarized in Section 2. Section 4 presents the detailed proposed methodology. The results and related discussion is presented in Section 4. The paper ends with conclusin and suggestions for future research in Section 5.

## 2 Related work

The prevalence of epilepsy, as one of the most common chronic neurological diseases, and the basic principle of EEG as a diagnostic test in epilepsy are discussed. As manual inspection of EEG recordings by clinicians is time consuming, subjective and hard to scale, automated seizure recognition is an active research field that has seen many different methods such as classical machine learning, deep learning, recurrent sequence modeling, and more recently attention-based architectures.The comprehensive review captures: traditional machine learning methods for EEG analysis, deep learning methods for EEG analysis, Transfromer and Self attention architectures, Graph Neural Network architectures and State Space and EEG foundation models mechanisms in EEG processing. The goal is to place an attention-enhanced hybrid CNN-BiLSTM framework for multiclass seizure recognition in this ongoing stream and to determine where it fits to solve the problem.

1. **Traditional machine learning approaches**
  Early automated systems for seizure detection used a two-stage framework: handcrafted feature extraction followed by a shallow (single or few layers) classifiers. The families can be categorized into three types of features. The statistical and linear approaches, including SVM and linear discriminant analysis, are widely adopted as low-cost models which establish direct connections between EEG features extracted from the data and class labels, and are thus applicable in realtime or hardware-limited systems. Distance based methods (e.g., *k*-nearest neighbors) classify a sample using similarity in feature space. Tree-based and ensemble methods such as random forests are robust to noisy or high dimensional features.
  The use of discrete-time complex wavelet transform (DTCWT) along with features derived by FFT and a least-squares SVM (LS-SVM) classifier was done by Al-Salman et al. (2019) and achieved an average accuracy of 97.7% on the Bonn EEG database. A different approach was used by Albaqami et al. (2023) who first converted the EEG signals into images with the help of the wavelet function before classification, reporting accuracies ranging from 98.97% to 99.5% over a number of class combinations for the Bonn dataset. In addition to the feature-engineering and shallow-classifier pipelines, Nafea and Ismail (2022) presented a systematic review and summarization of these pipelines, and Nahzat and Yaganoglu (2021) compared several classical ML algorithms following PCA-based dimensionality reduction. A more general system of feature extraction and shallow classifiers for detecting seizures is outlined.
  Although solid accuracy is reported on a set of well curated benchmark data sets like Bonn, traditional ML approaches have two architectural disadvantages that drive the transition to deep learning: (1) performance requires well-designed handcrafted features, which are not necessarily transferable across different recording montages or sampling rates or patient populations, etc., and (2) most pipelines are tested on binary or small multiclass data sets, with limited evidence of effectiveness on larger, more diverse, multiclass seizure-type data sets.
2. **Deep learning for EEG analysis**
  Later research focused on CNNs directly on EEG signals or on their time-frequency representations to minimize the need for manual feature engineering. There are two main ways of inputting the signals to the CNN, either as 1D-CNNs applied to raw or minimally filtered EEG waveforms, or 2D-CNNs applied to spectrograms, scalograms, or wavelet transforms, which represent the signals as image-like representations.
  Sadam and Nalini (2024) suggested a hybrid CNN using the scalogram transformation of EEG segments prior to classification, achieving good results for standard benchmarks. A recent study in 2025 by efficientnet2025svm introduced an EfficientNet-B0 CNN backbone and SVM classifiers in an ensemble framework using a voting strategy, using the normalized short-time Fourier transform (STFT) and channel-correlation features to reach an accuracy of 90.1% to 94.5% on the CHB-MIT dataset for the pre-ictal vs. inter-ictal classification with varying analysis window lengths. In other work (Hassan et al., 2022), a CNN was exclusively trained as an automatic feature extractor and the extracted features were passed to a conventional classifier afterwards, while the features were filtered based on the relevance of the information (e.g., using mutual information) to preserve the interpretability and less data requirement of the conventional classifiers. A related study that employs tunable-Q wavelet transform (TQWT) sub-band decomposition with fuzzy-entropy features and a deep classifier (Bhattacharyya et al., 2017) also shows comparable performance with feature-engineered baselines.
  Overall, these works have shown that CNN models can be trained directly on EEG with no hand-crafting of features, but that fully convolutional models are fundamentally restricted in their ability to capture the long-range temporal structure in seizure evolution over time, which is addressed by recurrent and hybrid models.
3. **Transformer and self-attention-only architectures**
  A large number of studies eliminate recurrence altogether and instead use self attention or full. blocks of transformers, due to the ability of the transformer to capture long-range dependencies Without a sequential bottleneck and by the interpretability provided by attention-weight visualization.
  In time-frequency analysis, Afzal et al. (2025) used a vision-transformer (ViT) architecture to perform the analysis. The electrographic classification of seizures using intracranial EEG in the form of a spectrogram representation, and Compared to ResNet-50, standard 2D CNNs and time-series transformers. Prior to the application of attention (AFTA) mechanism, the approach that was adopted was pretraining with an unlabeled EEG dataset. Using Adaptive Frequency Filtering for fine-tuning for seizure prediction and classification. Module fused with temporal attention to reject noise and artifact corruption. These works In this paper, we present attention-only backbones that excel in seizure-classification performance while showing attention. Eliminating the recurrent branch completely.
4. **Graph neural network architectures**
  A different type of job uses the EEG as a graph, rather than as an image or just a time series. electrodes as nodes and functional or structural connectivity as connections between pairs of nodes. The nature of epilepsy seizures is network. Li et al. (2024) proposed a multi-branch graph A multi-head attention network with a convolutional architecture (MGCNA) for childhood seizure detection, and Leveraging several graph learning strategies and spatial and temporal dependency modeling. Leveraging graph-based deep learning, Agaronyan et al. (2025) have predicted seizures in stereo-EEG. In patients with epilepsy, there is a freedom of the disease. Kotoge et al. (2025) proposed the model of EEG as a A two-stream Mamba architecture coupled with a graph transformer is employed to process the sequence of time-evolving graphs to produce a graph. A convolutional network that yielded up to 23% improvement in AUROC and 30% in F1 score.
5. **State-space (Mamba) and EEG foundation models**
  The current architecture family is based on a new concept, which replaces the convolution and attention with selective. Most commonly used state-space models (SSMs) which scale linearly, not quadratically, such as Mamba. with sequence length. Gui et al. (2024) have introduced EEGMamba, which is a combination of spatial-adaptive and temporal gating. A bidirectional Mamba block and a task-aware mixture-of-experts layer are used for convolution. Tested on eight public EEG databases. Tegon et al. (2025) developed Foundational EEG Mamba + Bidirectional Architecture (FEMBA), which is a Answer the question. The bidirectional Mamba foundation model is pre-trained using more than 21,000 h of unlabeled clinical data. EEG comes close to transformer performance with much lower computational cost.
  A number of studies have recently investigated the use of attention enhanced deep learning techniques for automatic epileptic seizure classification with EEG signals. A novel filter-bank multi-view and attention-based neural network was proposed (Feng et al., 2026), which decomposes the raw EEG signals into several frequency sub-bands by a learnable filter bank, and extracts the multi-scale frequency-spatial features by multi-branch group convolution. To highlight informative time windows and frequency bands, the temporal dependencies are further modeled using bidirectional long short-term memory (BiLSTM) with an attention mechanism. The model's performance on the TUSZ v1.5.2 dataset was overall score of 0.7105, weighted F1-score of 0.8314, and Cohen's kappa of 0.6345, with 3.22%, 1.42%, and 2.87% higher, respectively, than the performance of FBCNet. The model also showed superior performance on CHB-MIT dataset which enabled the effectiveness of multi-view frequency analysis, multi-scale feature extraction and attention-based temporal modeling for the robust classification of EEG signals. Hasan and Islam (2026) proposed an Adaptive Frequency Band Attention (AFBA)-guided 2D CNN-BiLSTM framework for EEG-based emotion recognition. The approach adaptively weights different EEG frequency bands and combines spatial-spectral feature extraction using CNN with temporal dependency modeling through BiLSTM. Evaluated on the DEAP and SEED datasets, the model achieved up to 97.2% valence and 98.1% arousal accuracy on DEAP, demonstrating the effectiveness of adaptive frequency-band attention and temporal modeling for EEG classification. The Table 1 summarizes the various methodologies for EEG classification.

**Table 1:** Summary of existing EEG-based seizure classification approaches.

References	Architecture/ method	Dataset	Task/classes	Main outcome/observation
Palanisamy and Rengaraj (2024)	FCM–PS–LSTM	University of Bonn EEG	Seizure classification	Reported an accuracy of 98.00%, demonstrating the effectiveness of combining feature selection with recurrent temporal modeling.
Kunekar et al. (2024)	Machine Learning and Deep Learning Framework	Bonn EEG	Seizure classification	Achieved 97.1% accuracy, 96% precision, 90% recall, and 93% F1-score.
Kanamaneni et al. (2025)	Convolutional Neural Network (CNN)	EEG Seizure Dataset	Seizure classification	Reported an accuracy of 84.2%. The study demonstrates the applicability of CNN-based feature learning for EEG classification.
Al-Marzouki (2025)	BiLSTM	Kaggle Epileptic Seizure Recognition	Multiclass seizure classification	Achieved 98.70% accuracy, 99.00% precision, 98.40% recall, and 98.70% F1-score.
Wang et al. (2026)	Time–Frequency Cross-Attention Network	Bonn + HMS Kaggle EEG	Seizure classification	Reported accuracies of 93.63% and 96.15% on the evaluated datasets, demonstrating the benefit of time–frequency attention modeling.
Afsari et al. (2026)	Six-Channel BiLSTM	Clinical EEG	Seizure detection	Achieved 87.75% accuracy and 77.3% recall, highlighting the challenges associated with clinical multi-channel EEG data.
Feng et al. (2026)	Filter-bank multi-view and attention-based neural network	TUSZ v1.5.2; CHB-MIT	Multiclass seizure classification	Combined learnable filter-bank decomposition, multi-scale feature extraction, BiLSTM and attention. Reported overall score of 0.7105, weighted F1-score of 0.8314, and Cohen's kappa of 0.6345 on TUSZ.
Hasan and Islam (2026)	Adaptive Frequency Band Attention-guided 2D CNN–BiLSTM	DEAP; SEED	EEG emotion recognition	Adaptively weights frequency bands and combines CNN-based spatial–spectral extraction with BiLSTM temporal modeling. Achieved up to 97.2% valence and 98.1% arousal accuracy on DEAP.
Gui et al. (2024)	EEGMamba: Bidirectional Mamba + Mixture-of-Experts	Eight public EEG datasets	Multi-task EEG analysis, including seizure detection	Achieved state-of-the-art performance on the majority of evaluated tasks while providing linear sequence modeling through state-space mechanisms.
Tegon et al. (2025)	FEMBA: Bidirectional Mamba foundation model	Large-scale clinical EEG	Multiple downstream tasks, including neonatal seizure detection	Used more than 21,000 h of self-supervised pretraining followed by fine-tuning. Reported near-transformer accuracy with substantially lower computational cost.

### 2.1 Synthesis and research gap

From the literature reviewed, there are four definite patterns. The first is that there is a natural progression of pipeline architectures: handcrafted-feature and shallow classifiers, through to end-to-end CNNs, to CNN-recurrent hybrids, and finally to attention-augmented hybrids, which tend to improve accuracy and reduce the need for manual feature engineering as they go along. Second, bidirectional recurrent layers (BiLSTM/BiGRU) always outperform their unidirectional rivals and CNN-only baselines because they are able to take into account the bidirectional temporal context. Third, attention mechanisms, either channel/spatial (CBAM) or multi-head self-attention or dual-attention, have been shown to give an additional accuracy boost when added to CNN-BiLSTM/BiGRU backbones, and also enhance interpretability, which is becoming more and more relevant for clinical translation. Thirdly, most of the high accuracy rates are reported for binary or small multiclass (2–3 class) problems on small single-source benchmark datasets (typically Bonn EEG database or CHB-MIT); comparatively less studies are available on CNN-BiLSTM-attention architectures for granular multiclass seizure-type recognition and even fewer studies report cross-patient or cross-dataset validation. This is where the novel idea of combining CNN and BiLSTM in a hybrid framework, with an added attention mechanism is expected to cater: it is demonstrated that the combination of CNN and BiLSTM is complementary in the modeling of spatial-temporal features, while attention is proven to be accurate and interpretable, both of which are explicitly extended to multiclass seizure recognition.

## 3 Methodology

The proposed framework is designed to classify epileptic seizure events from EEG signals using a hybrid deep learning architecture that combines convolutional neural networks (CNN), bidirectional long short-term memory (BiLSTM), and multi-head self-attention. The complete workflow consists of five major stages: (i) EEG signal acquisition and preprocessing, (ii) data augmentation, (iii) feature extraction through CNN layers, (iv) temporal dependency learning using BiLSTM enhanced with self-attention, and (v) seizure classification using fully connected layers.

### 3.1 Dataset

This study utilized the Kaggle Epileptic Seizure Recognition dataset, which is a pre-processed and reshaped version of the benchmark EEG time-series data. The dataset comprises 11,500 EEG recordings, each consisting of 178 temporal samples recorded at a sampling frequency of 178 Hz, yielding one-second signal epochs. Each recording is originally labeled into five classes representing different neural activity states. The original class labels were transformed into numerical representations using label encoding to facilitate model training. The encoding process mapped the five EEG classes to integer values ranging from 0 to 4 while preserving the original class distribution. As shown in Table 2, each class contains 2,300 EEG segments, resulting in a balanced dataset comprising 11,500 samples. No class merging or relabeling was performed; therefore, the proposed framework was evaluated as a five-class classification problem. The class labels 1, 2, 3, 4, 5 represent:

**Table 2:** Class distribution after label encoding and corresponding EEG recording conditions.

Original class	Encoded label	Samples	EEG recording condition
1	0	2,300	Seizure activity: EEG signals were recorded during an active seizure event.
2	1	2,300	Tumor region: EEG signals were recorded from the brain region where the tumor was located.
3	2	2,300	Healthy brain region: EEG signals were recorded from the healthy brain area, with the tumor location identified separately.
4	3	2,300	Eyes closed: EEG signals were recorded while the subject's eyes were closed.
5	4	2,300	Eyes open: EEG signals were recorded while the subject's eyes were open.
**Total**	–	**11,500**	–

Bold indicates total samples (all classes together).

The five classes represent different physiological and/or recording conditions. Two classes are used to represent healthy resting state activity, one for when the subject is awake with open eyes (Class 5) and one when awake with closed eyes (Class 4). The other two classes are from tumor patients with activity in an untreated area of the brain (Class 3) and in the tumor area itself (Class 2). The other class, Class 1, is activity recorded while an active seizure event occurs. Only Class 1 is considered to have true epileptic activity and all other five classes are grouped together as the non-seizure group for this study.

The dataset was partitioned into training and testing subsets using an 80:20 split ratio to ensure a balanced evaluation of the proposed model. To preserve the original class distribution across both subsets, stratified sampling was applied during the splitting process. A fixed random seed *random*_*state* = 42 was used to guarantee reproducibility of the results.

The original class labels were transformed into numerical representations using label encoding. This process mapped the five EEG classes to integer values ranging from 0 to 4 while preserving the original class distribution. No class merging or relabeling was performed; therefore, the study was conducted as a five-class classification problem comprising 11,500 EEG segments equally distributed across the five categories.

### 3.2 EEG signal preprocessing

For the reduction of both physiological noise and instrumentation noise, while preserving the diagnostically relevant frequency content, every EEG epoch was subjected to a two-step preprocessing procedure: frequency domain filtering and amplitude normalization. Because of the sensitivity of the raw EEG to various contaminating factors, including baseline wander from electrode drift and respiration, high frequency electromyographic (muscle) interference and power-line interference, subtle and clinically significant EEG abnormalities, including spikes, sharp waves and rhythmic discharges, can be masked. Unless addressed, these artifacts add noise to the raw signal a deep learning model could potentially include as a part of its learned class label, leading to poorer generalization to new recordings. The preprocessing pipeline was therefore designed not only as a denoising step, but rather as a principled transformation that will ensure that the signal is presented in a standardized format to the network's convolutional filters, allowing the network to operate on a fixed, physiologically meaningful representation of the signal across all samples, subjects, and recording sessions.

#### 3.2.1 Bandpass filtering

A fifth-order Butterworth bandpass filter was designed with cutoff frequencies of 0.5 Hz and 40 Hz, corresponding to the clinically relevant delta-through-gamma range in which epileptiform activity is typically expressed. The cutoff frequencies were normalized with respect to the Nyquist frequency (half the sampling rate, i.e., 89 Hz), and the filter coefficients were computed as:


$$\begin{array}{l} W_{\text{low}}=\frac{f_{\text{low}}}{f_{s}/2},\;W_{\text{high}}=\frac{f_{\text{high}}}{f_{s}/2} \end{array}$$

where *f*_low_ = 0.5 Hz, *f*_high_ = 40 Hz, and *f*_*s*_ = 178 Hz.

The filtered signal is obtained using a Butterworth band-pass filter as


$$\begin{array}{l} x_{f}\left(t\right)=\text{Butterworth}\left(x\left(t\right)\right) \end{array}$$

where *x*(*t*) represents the original EEG signal and *x*_*f*_(*t*) denotes the filtered EEG signal.

To eliminate the phase distortion introduced by standard IIR filtering, the filter was applied using zero-phase forward-backward filtering (filtfilt), which processes the signal in both temporal directions and effectively doubles the filter order while preserving the timing of transient events such as spike-wave discharges. Any missing or non-finite sample values were replaced with zero prior to filtering to ensure numerical stability.

#### 3.2.2 *Z*-score normalization

Each filtered epoch was normalized to zero mean and unit variance using *z*-score normalization:


$$x_{\text{normalized}}=\frac{x-\mu }{\sigma }$$

where μ and σ denote the mean and standard deviation of the individual epoch, and ϵ = 1 × 10^−8^ is a small constant introduced to prevent division by zero. Per-sample (rather than global) normalization was adopted to compensate for inter-trial amplitude variability arising from differences in electrode impedance and montage across recordings, ensuring that the network learns morphological rather than amplitude-driven discriminative features. This standardization ensures equal contribution of all frequency components during model training and improves gradient flow in deep networks.

### 3.3 Data augmentation

To improve the generalization capability of the proposed network and reduce overfitting caused by the moderate dataset size, an on-the-fly data augmentation strategy was applied to the training set after preprocessing. For each original EEG signal, two additional synthetic variants were generated.

#### 3.3.1 Additive Gaussian noise


$$\begin{array}{l} x_{\text{aug}}=x+N\left(0,0.01^{2}\right) \end{array}$$

where $N\left(0,0.01^{2}\right)$ denotes zero-mean Gaussian noise with a standard deviation of 0.01. This augmentation simulates low-level sensor and environmental noise while preserving the underlying seizure-related signal morphology.

##### 3.3.1.1 Random amplitude scaling


$$\begin{array}{l} x_{\text{aug}}=x\cdot \alpha \end{array}$$

where


$$\begin{array}{l} \alpha \sim U\left(0.9,1.1\right) \end{array}$$

and U(0.9,1.1) represents a uniform distribution. This transformation emulates natural inter-subject amplitude variability commonly observed in EEG recordings.

The augmentation process effectively balances the dataset by generating noise-perturbed and amplitude-scaled versions of each original signal. Consequently, a more robust and balanced training distribution was obtained without modifying the semantic meaning of the class labels.

### 3.4 Label encoding and dataset partitioning

A LabelEncoder was used to convert the categorical class labels into integer indices. The data was then split into training and held-out test sets with a 80/20 stratified split, stratified on the encoded class labels to ensure the class distribution in both sets was not biased toward any particular class. The same random seed was used for all the runs to guarantee the reproducibility. All one-dimensional signals were reshaped to rank 3 tensors (samples, timesteps, 1) to match the input dimensions of the one-dimensional CNN layers. The Epileptic Seizure Recognition dataset consists of 11,500 EEG segments, each represented by 178 temporal features. The target variable contains five balanced classes with 2,300 samples per class. Prior to model training, the class labels were transformed into numerical representations using label encoding, resulting in five distinct classes indexed from 0 to 4. Table 3 summarizes the class-wise distribution of samples before and after train–test splitting.

**Table 3:** Class distribution after train–test splitting.

Class	Label	Total	Train	Test
1	0	2,300	1,840	460
2	1	2,300	1,840	460
3	2	2,300	1,840	460
4	3	2,300	1,840	460
5	4	2,300	1,840	460
Total	–	1,1500	9,200	2,300

The data set was split into training and test sets with stratified random sampling to assess the proposed model in an unbiased way. In particular, 80% of the samples were used for training while 20% were used for testing. Stratification was used to maintain the same class distribution for the two subsets, and the same random seed (42) was used to ensure the reproducibility of the experiments.

The resulting feature matrices had dimensions of 9,200 × 178 and 2,300 × 178 for the training and testing sets, respectively. Corresponding label vectors contained 9,200 and 2,300 samples. The preservation of class balance in both subsets facilitates robust learning and fair performance evaluation of the proposed epileptic seizure classification framework.

### 3.5 Proposed hybrid CNN-BiLSTM-attention architecture

We present a hybrid deep neural network combining multi-scale temporal modeling, bidirectional recurrent networks, and multi-head self-attention within a single end-to-end trainable network that can well capture the multi-scale temporal properties of EEG signals. This design rationale is comprised of three points: (i) one-dimensional convolutional layers are appropriate to extract local translation-invariant morphological patterns like spikes and sharp waves; (ii) the BiLSTM layer is useful to capture long-range temporal dependencies in both directions, because ictal activity is non-stationary and evolves over time; and (iii) the multi-head self-attention mechanism enables the network to selectively focus on the most discriminative time steps in a sequence, regardless of their temporal distance from one another. The overall architecture is shown in Figure 1 and formalized mathematically in the following subsections.

**Figure 1:**
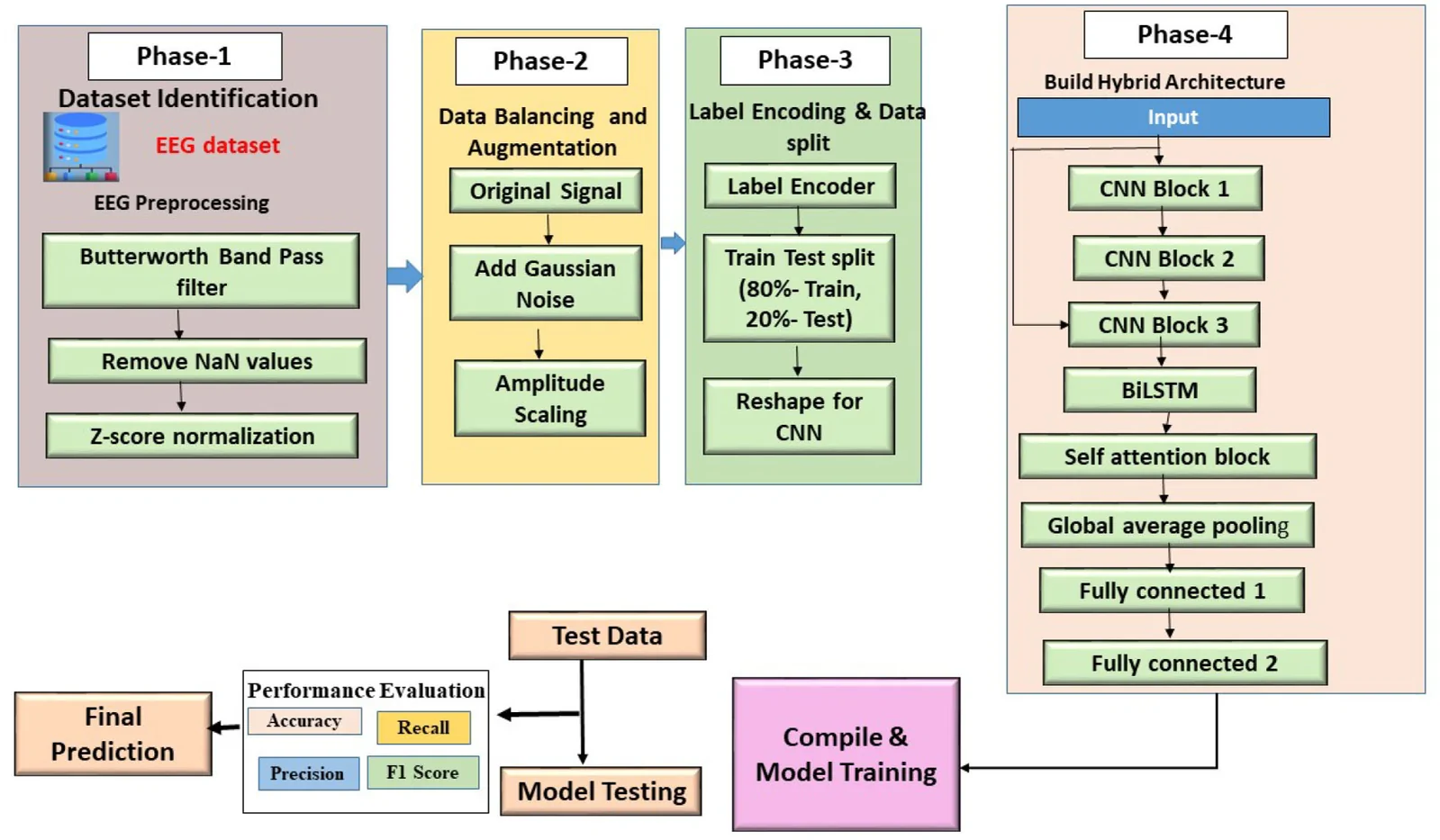
The proposed model.

The proposed hybrid deep learning framework fuses three models, such as CNN, BiLSTM and multi-head self-attention (MHSA), to accurately classify epileptic seizures. The CNN extracts local spatial properties from EEG signals and the Bi-LSTM captures the long-term temporal properties, both forward and backward. Most informative temporal regions are then selected using the multi-head self-attention (MSA) mechanism and can be adaptively weighted before final classification.

#### 3.5.1 Architecture overview

Let *x* ∈ ℝ^*T*×1^ denote a preprocessed, normalized univariate EEG epoch of length *T* time steps. The proposed network *f*_θ_(·) maps *x* to a class probability distribution ŷ ∈ ℝ^*C*^ over *C* target classes through four sequential functional stages:


$$\begin{array}{l} f_{\theta }\left(x\right)=\text{Softmax}(\text{Dense}(\text{MHSA}(\text{BiLSTM}(\text{CNN}\left(x\right))))) \end{array}$$

where CNN(·) denotes the convolutional feature extractor, BiLSTM(·) the bidirectional recurrent encoder, MHSA(·) the multi-head self-attention refinement stage, and Dense(·) the fully connected classification head. Figure 1 depicts this pipeline schematically.

#### 3.5.2 Convolutional feature extraction block

The convolutional front-end consists of three sequential one-dimensional convolutional blocks that progressively extract local waveform features at increasing levels of abstraction, while reducing temporal resolution and controlling overfitting through pooling and dropout. Each convolution operation at layer *l* is defined as:


$$\begin{array}{l} h_{i}^{\left(l\right)}=\text{ReLU}\left(\overset{K_{l}}{\underset{k=1}{\sum }}w_{k}^{\left(l\right)}h_{i+k-1}^{\left(l-1\right)}+b^{\left(l\right)}\right) \end{array}$$

where *h*^(*l*−1)^ is the input feature map to layer *l*, $w_{k}^{\left(l\right)}$ and *b*^(*l*)^ are the learnable kernel weights and bias, and *K*_*l*_ is the kernel size at layer *l*. Batch normalization is applied after each convolution to stabilize training:


$$\begin{array}{l} \text{BN}\left(h\right)=\gamma \cdot \frac{h-\mu_{B}}{\sqrt{\sigma_{B}^{2}+\epsilon }}+\beta \end{array}$$

where $\mu_{B}$ and $\sigma_{B}^{2}$ are the batch mean and variance, and γ, β are learnable scale and shift parameters. The configuration of each convolutional block is summarized in Table 4.

**Table 4:** Configuration of the convolutional feature extraction block.

Block	Filters	Kernel	Activation	Pooling	Dropout
Conv block 1	32	5	ReLU	MaxPool(2)	0.2
Conv block 2	64	3	ReLU	MaxPool(2)	0.3
Conv block 3	128	3	ReLU	–	0.3

#### 3.5.3 Bidirectional LSTM encoder

The output feature sequence from the convolutional block, $H_{cnn}=\left\{h_{1},h_{2},\ldots ,h_{T^{\prime }}\right\}$, is passed to a Bidirectional LSTM layer to model temporal dependencies in both forward and backward directions. At each time step *t*, the forward and backward LSTM cells compute their hidden states as:


$$\begin{array}{l} \vec{h_{t}}=\text{LST}{\text{M}}_{fwd}\left(h_{t},{\vec{h}}_{t-1}\right),\;\overset{⃖}{h_{t}}=\text{LST}{\text{M}}_{bwd}\left(h_{t},{\overset{⃖}{h}}_{t+1}\right) \end{array}$$

with the internal LSTM cell update governed by the standard gating equations:


$$\begin{array}{l} f_{t}=\sigma \left(W_{f}\left[h_{t-1},x_{t}\right]+b_{f}\right)\\ i_{t}=\sigma \left(W_{i}\left[h_{t-1},x_{t}\right]+b_{i}\right)\\ {\tilde{c}}_{t}=\tanh\left(W_{c}\left[h_{t-1},x_{t}\right]+b_{c}\right)\\ c_{t}=f_{t}⊙c_{t-1}+i_{t}⊙{\tilde{c}}_{t}\\ o_{t}=\sigma \left(W_{o}\left[h_{t-1},x_{t}\right]+b_{o}\right)\\ h_{t}=o_{t}⊙\tanh\left(c_{t}\right) \end{array}$$

where σ(·) is the sigmoid activation, ⊙ denotes element-wise multiplication, and *f*_*t*_, *i*_*t*_, *o*_*t*_, and *c*_*t*_ represent the forget, input, output gates, and cell state, respectively. The final BiLSTM representation at each time step is obtained by concatenating the forward and backward hidden states:


$$\begin{array}{l} H_{bilstm,t}=\left[\vec{h_{t}};\overset{⃖}{h_{t}}\right]\in {\mathbb{R}}^{256} \end{array}$$

Because return_sequences=True is used, the full sequence $H_{bilstm}\in {\mathbb{R}}^{T^{\prime }\times 256}$ is retained (rather than only the final hidden state) to preserve the complete temporal context required by the subsequent attention layer.

#### 3.5.4 Multi-head self-attention refinement

To allow the network to dynamically emphasize the most seizure-relevant time steps regardless of their temporal distance, a multi-head self-attention mechanism is applied to *H*_*bilstm*_. For a single attention head, the scaled dot-product attention is computed as:


$$\begin{array}{l} \text{Attention}\left(Q,K,V\right)=\text{softmax}\left(\frac{QK^{⊤}}{\sqrt{d_{k}}}\right)V \end{array}$$

where $Q=H_{bilstm}W^{Q}$, $K=H_{bilstm}W^{K}$, and $V=H_{bilstm}W^{V}$ are the query, key, and value projections, and *d*_*k*_ = 32 is the key dimension used for scaling. With *h* = 4 parallel attention heads, the outputs are concatenated and linearly projected:


$$\begin{array}{c} \text{MHSA}\left(H\right)=\left[\text{hea}{\text{d}}_{1};\text{hea}{\text{d}}_{2};\text{hea}{\text{d}}_{3};\text{hea}{\text{d}}_{4}\right]W^{O},\\ \text{hea}{\text{d}}_{j}=\text{Attention}\left(QW_{j}^{Q},KW_{j}^{K},VW_{j}^{V}\right) \end{array}$$

The attention output is then combined with the original BiLSTM representation via a residual connection, followed by layer normalization, to stabilize gradient flow and preserve the recurrent representation while allowing the attention mechanism to refine it:


$$\begin{array}{l} Z=\text{LayerNorm}(H_{bilstm}+\text{MHSA}\left(H_{bilstm}\right)) \end{array}$$

The configuration of the attention mechanism is summarized in Table 5.

**Table 5:** Configuration of the multi-head self-attention block.

Parameter	Value
Number of heads (*h*)	4
Key dimension (*d*_*k*_)	32
Residual connection	Yes
Normalization	Layer normalization

#### 3.5.5 Global feature aggregation and classification

The attention-refined sequence *Z* ∈ ℝ^*T*^′^^ × 256 is aggregated across the temporal dimension via global average pooling to obtain a fixed-length representation independent of sequence length:


$$\begin{array}{l} z_{pool}=\frac{1}{T^{\prime }}\overset{T^{\prime }}{\underset{t=1}{\sum }}Z_{t} \end{array}$$

This pooled vector is passed through a fully connected layer with 256 units and ReLU activation, followed by dropout (rate 0.5), and finally a softmax output layer that produces the class probability distribution:


$$\begin{array}{l} {ŷ}_{c}=\frac{\exp\left(w_{c}^{⊤}z_{fc}+b_{c}\right)}{\sum_{j=1}^{C}\exp\left(w_{j}^{⊤}z_{fc}+b_{j}\right)},\;c=1,\ldots ,C \end{array}$$

where *z*_*fc*_ is the output of the dense layer and *C* is the number of target seizure/non-seizure classes. The complete layer-wise configuration of the proposed model is summarized in Table 6. Table 7 provides description of Hyper-parameters of the Proposed CNN–BiLSTM–MHSA Model.

**Table 6:** Complete layer-wise configuration of the proposed hybrid model.

Stage	Layer	Output units/filters
CNN block	Conv1D + BatchNorm + MaxPool + dropout(0.2)	32
	Conv1D + BatchNorm + MaxPool + dropout(0.3)	64
	Conv1D + BatchNorm + dropout(0.3)	128
Recurrent encoder	Bidirectional LSTM + dropout(0.4)	256 (128 × 2)
Attention	Multi-head self-attention (four heads)	256
	Residual add + LayerNorm	256
Pooling	Global average pooling	256
Classification head	Dense + ReLU + dropout(0.5)	256
	Dense + Softmax	*C*

**Table 7:** Training hyperparameters of the proposed CNN–BiLSTM–MHSA model.

Parameter	Value
Optimizer	Adam
Initial learning rate	0.001
Loss function	Sparse categorical cross-entropy
Maximum epochs	80
Batch size	64
Training-test split	80:20
CNN filters	32, 64, 128
Kernel sizes	5, 3, 3
Pooling size	2
BiLSTM units	128
Attention heads	4
Key dimension	32
Dense layer neurons	256
Dropout rates	0.2, 0.3, 0.3, 0.5
Activation function	ReLU, Softmax
Early stopping patience	10 epochs
Learning rate reduction	Factor = 0.5
Validation monitor	Validation loss
Model saving format	HDF5 (.h5)

#### 3.5.6 Training objective

The complete network is trained end-to-end by minimizing the categorical cross-entropy loss between the predicted class distribution ŷ and the true one-hot encoded label *y*:


$$\begin{array}{l} L\left(\theta \right)=-\overset{C}{\underset{c=1}{\sum }}y_{c}\log\left({ŷ}_{c}\right) \end{array}$$

Optimization is performed with the help of Adam optimizer with an starting learning rate of 1 × 10^−3^. Early stopping (patience of 10 epochs, monitored on validation loss) and a reduce-on-plateau learning rate schedule (decay factor of 0.5 after 5 epochs of stagnation) are employed to mitigate overfitting and improve convergence stability.

### 3.6 Training details

The proposed CNN–BiLSTM–MHSA model was implemented using TensorFlow and Keras. Pre-processing of the EEG signals involved a fifth-order Butterworth bandpass filter (0.5–40 Hz) and *Z*-score normalization. A data augmentation technique based on Gaussian noise injection and random amplitude scaling was used to increase the model's generalization. This data set was split into two sets: a training set and a testing set, with a ratio of 0.8 to 0.2.The data set was divided into two sets as training set and testing set with a ratio of 0.8:0.2 and the input signals are reshaped into one dimensional tensors of size *L*×1. To mitigate overfitting and enhance the generalization capability of the proposed attention-enhanced CNN-BiLSTM framework, multiple regularization strategies were employed. Dropout regularization (rate = 0.30) was applied to key dense and recurrent layers to reduce feature co-adaptation, while data augmentation using controlled Gaussian noise and random amplitude scaling was performed exclusively on the training set after the train-test split to prevent data leakage. In addition, early stopping based on validation loss was used to terminate training when performance ceased to improve, thereby avoiding overfitting to the training data. A stratified validation strategy was also adopted to ensure balanced class representation during training. The effectiveness of these measures is reflected in the stable convergence of training and validation loss and accuracy curves, indicating consistent learning behavior without significant overfitting.

The network is composed of three Conv1D layers with 32, 64 and 128 filters, followed by Batch Normalization, Max-Pooling (first two layers) and Dropout (0.2, 0.3, 0.3). The extracted features are then passed through a BiLSTM layer with 128 units and a Multi-Head Self-Attention module consisting of 4 attention heads, each having a key dimension of 32. The Global Average Pooling layer combines the learned features before classification with a fully connected 256 neuron layer using ReLU activation, dropout (0.5) and a Softmax output layer.

The model was optimized using Adam with a learning rate of 1 × 10^−3^, and sparse categorical cross entropy loss function. The number of epochs trained was capped at 80 and the batch size was set to 64. Other steps taken to avoid overfitting and convergence were early stopping (patience = 10) and ReduceLROnPlateau (factor = 0.5; patience = 5). The performance of models was measured by the parameters such as Accuracy, Precision, Recall, F1-score, ROC–AUC, confusion matrix and classification report. Algorithm 1 provides step by step Training Procedure of the Proposed CNN–BiLSTM–MHSA Mode.

Algorithm 1 Training procedure of the proposed CNN–BiLSTM–MHSA model. 
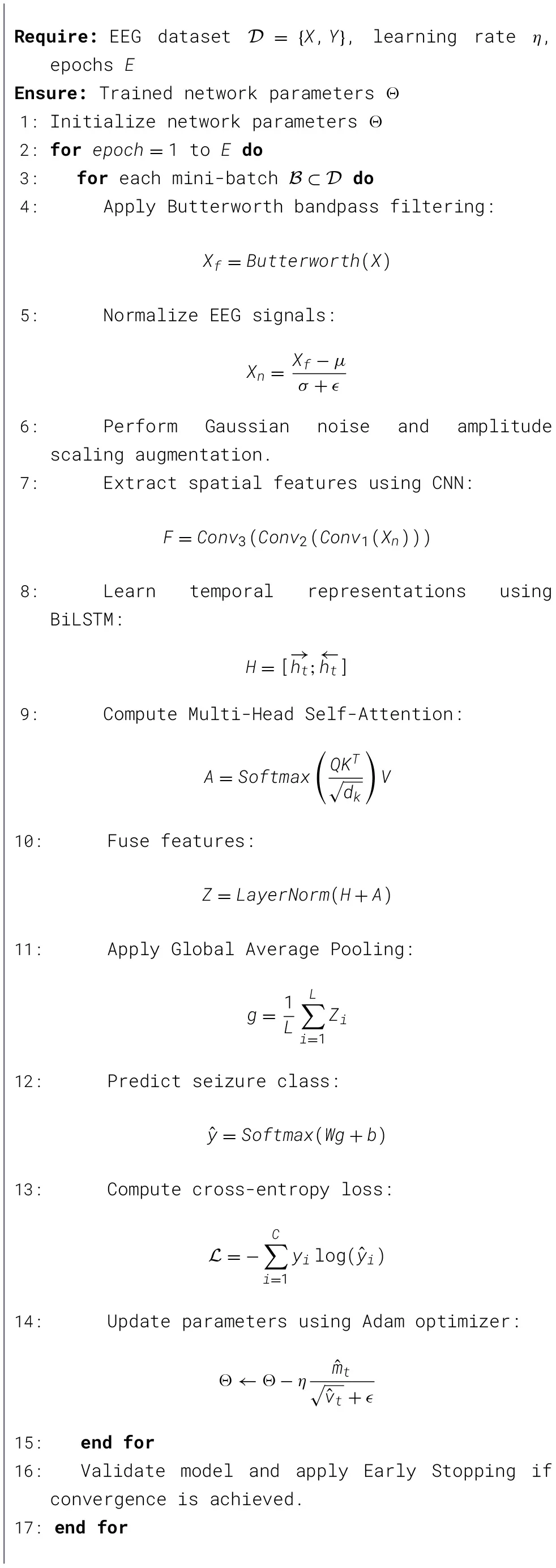

## 4 Results and discussion

The performance of the proposed CNN–BiLSTM–Multi-Head Self-Attention (CNN–BiLSTM–MHSA) model was evaluated using multiple quantitative and qualitative performance measures. Besides overall classification accuracy, Precision, Recall, F1-score, confusion matrix, Precision–Recall (PR) curves, feature visualization, attention analysis, and t-SNE were employed to assess the effectiveness of the proposed framework. The experiments were conducted using an 80:20 train-test split and optimized using the Adam optimizer with an initial learning rate of 10^−3^.

### 4.1 Training performance

Figure 2 illustrates the learning behavior of the proposed model. The training accuracy increased steadily from approximately 61% to nearly 99%, while the validation accuracy followed a similar trend and converged to approximately 99%. The close agreement between the training and validation curves indicates excellent generalization capability without significant overfitting.

**Figure 2:**
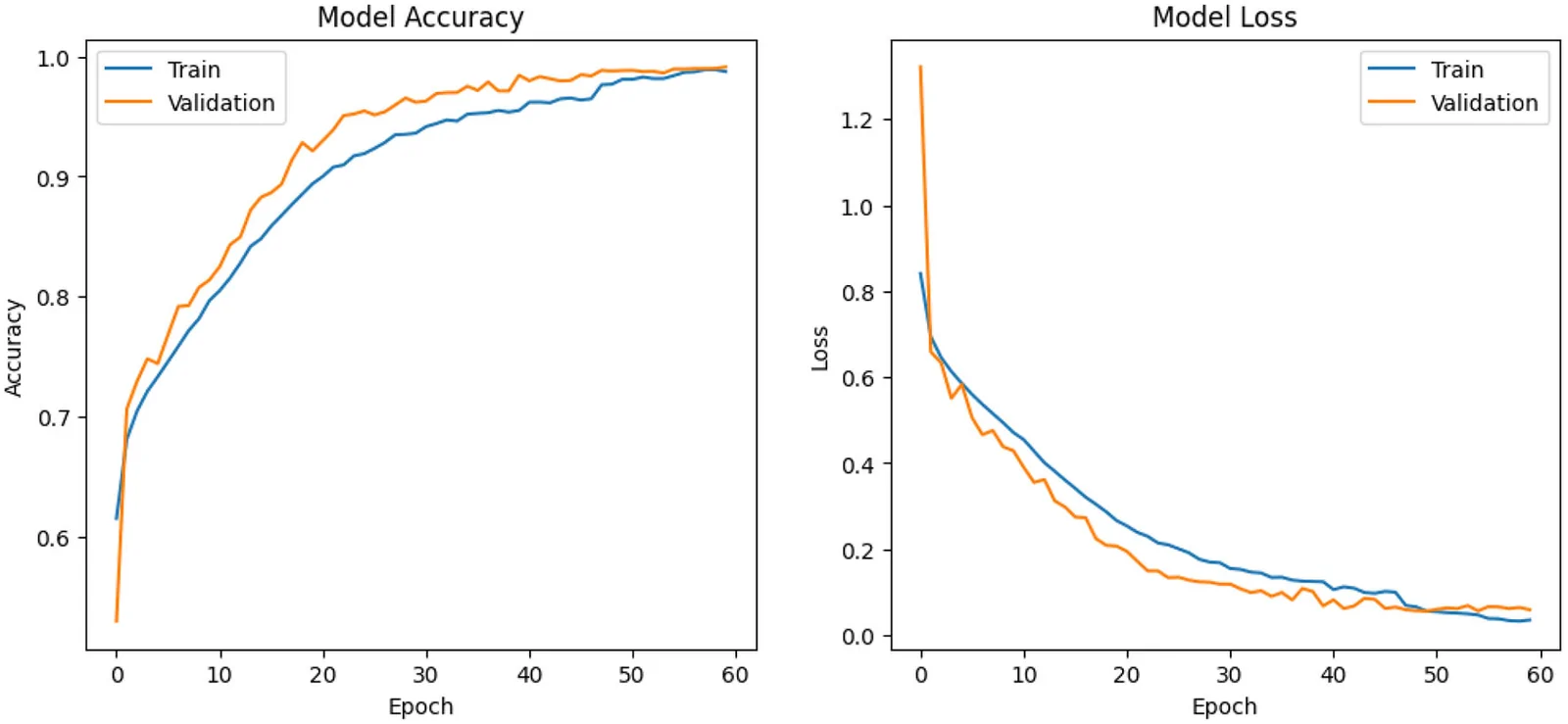
Training and validation accuracy and loss curves of the proposed CNN–BiLSTM–MHSA model.

Similarly, the training and validation losses decreased consistently throughout the optimization process, reaching values below 0.05 after convergence. The smooth convergence demonstrates the effectiveness of the adopted optimization strategy, including Early Stopping and ReduceLROnPlateau.

### 4.2 Confusion matrix analysis

The confusion matrix shown in Figure 3 demonstrates the classification capability of the proposed network. Most samples are correctly classified, resulting in strong diagonal dominance. Class 0 achieved perfect recognition, whereas only a few samples from Classes 1–4 were misclassified into neighboring categories because of their similar temporal characteristics.

**Figure 3:**
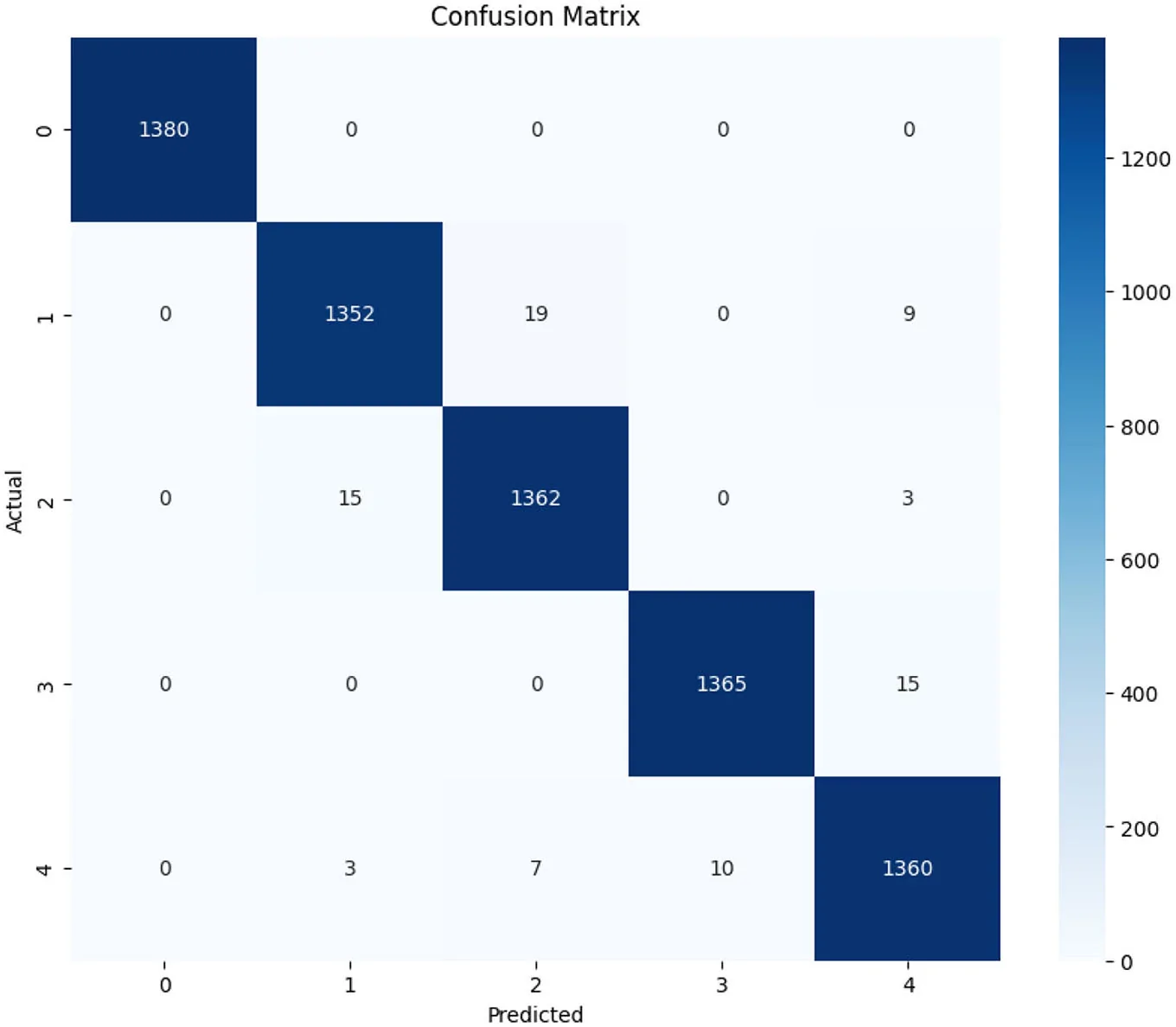
Confusion matrix obtained using the proposed CNN–BiLSTM–MHSA model.

### 4.3 Precision–recall analysis

Figure 4 presents the Precision–Recall curves for all seizure classes. The proposed model maintains precision values close to one over almost the complete recall range for all classes. Only a slight decrease is observed near complete recall, indicating a very small number of difficult samples. These results demonstrate the ability of the proposed network to simultaneously minimize false positives and false negatives.

**Figure 4:**
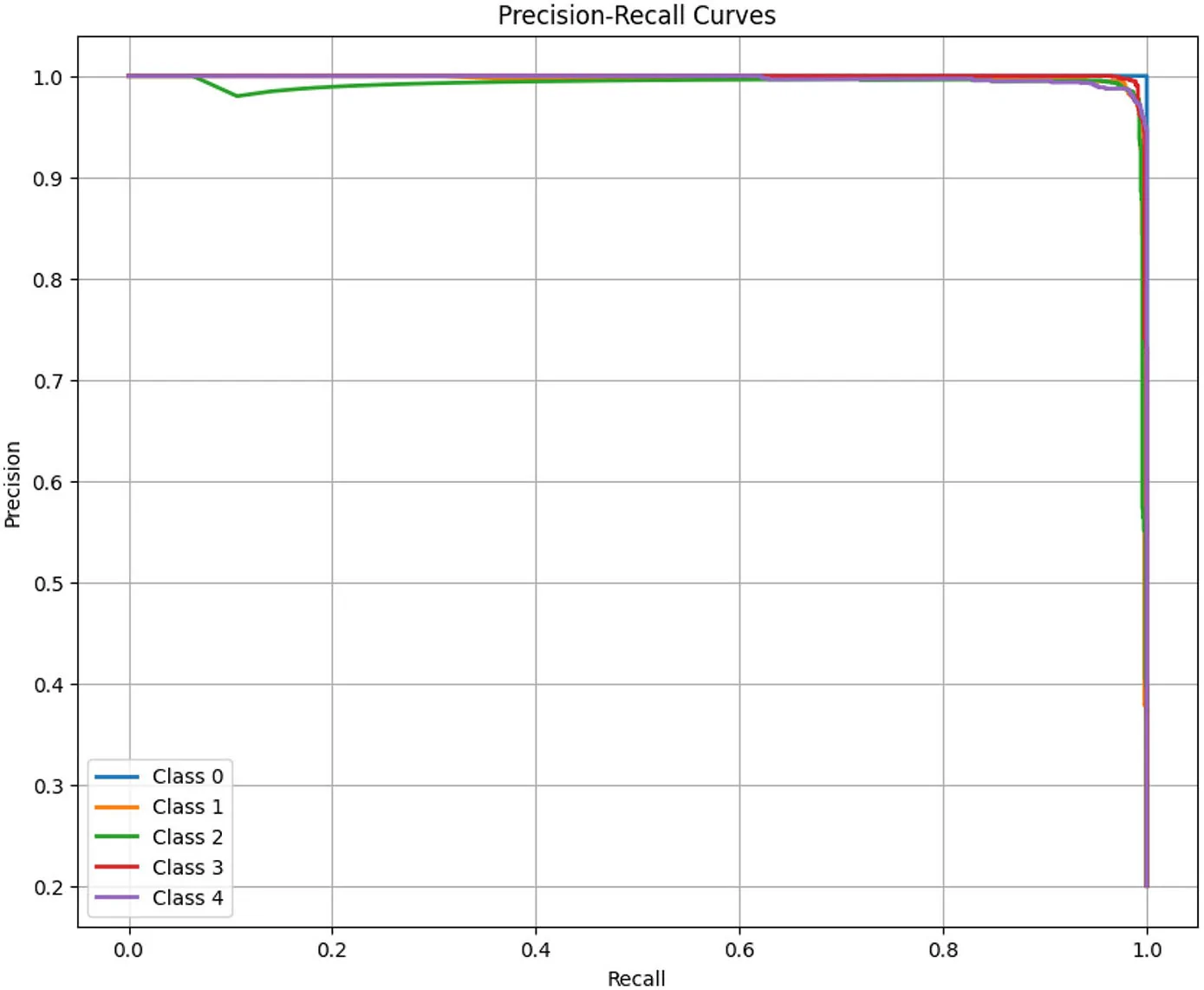
Precision–recall curves for the five EEG classes.

### 4.4 Per-class performance

The class-wise accuracies are illustrated in Figure 5. All five classes achieved classification accuracies greater than 98%, demonstrating the balanced performance of the proposed model across all seizure categories. Table 8 demonstrates classwise performance of the proposed model.

**Figure 5:**
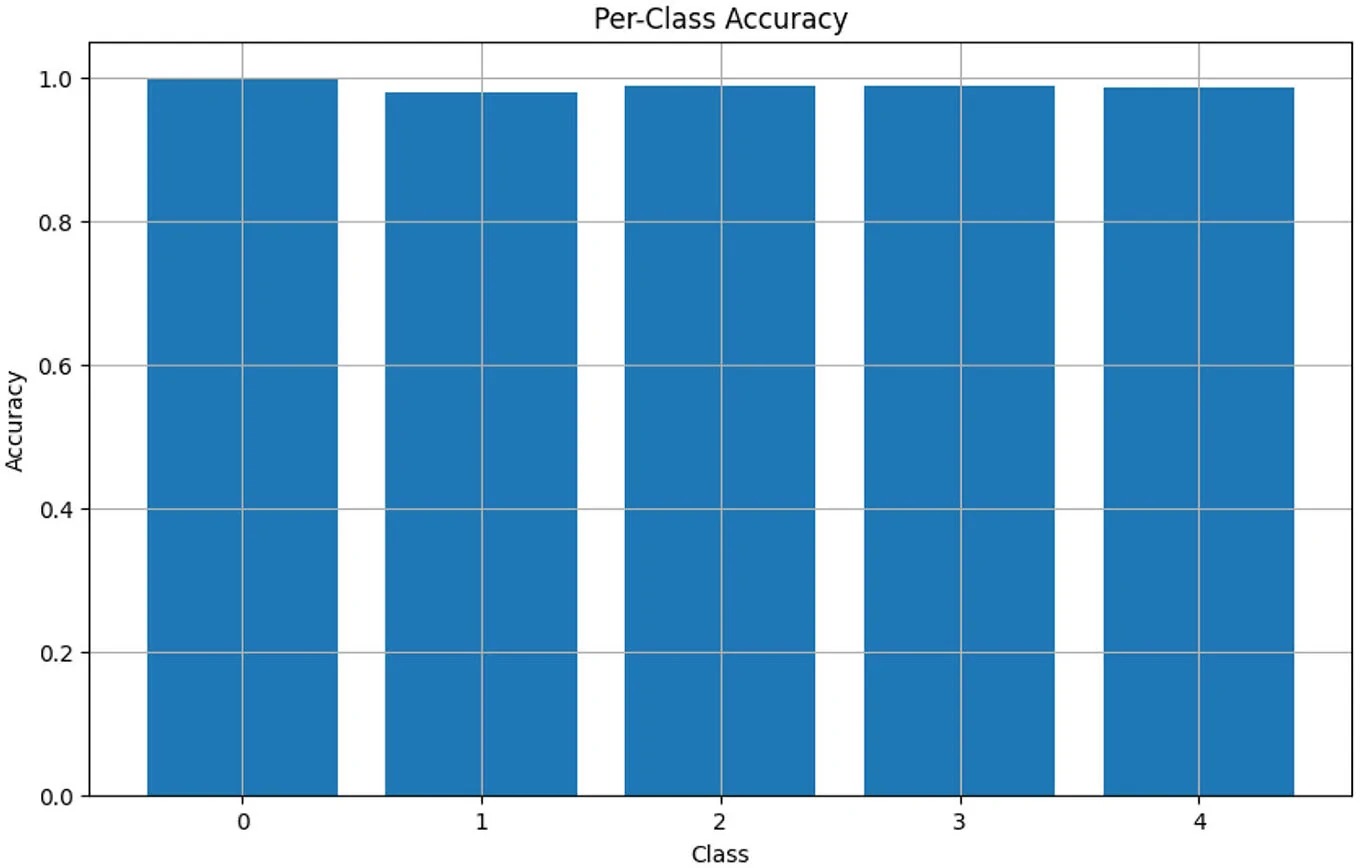
Per-class classification accuracy.

**Table 8:** Per-class performance of the proposed CNN–BiLSTM–MHSA model.

Class	TP	Precision (%)	Recall (%)	F1-score (%)	Accuracy (%)	Support
Class 0	1,380	100.00	100.00	100.00	100.00	1,380
Class 1	1,352	98.69	98.04	98.36	99.12	1,380
Class 2	1,362	98.12	98.70	98.41	99.13	1,380
Class 3	1,365	99.27	98.91	99.09	99.46	1,380
Class 4	1,360	98.69	98.55	98.62	99.28	1,380
**Macro average**	–	**98.95**	**98.84**	**98.90**	**99.40**	6,900
**Weighted average**	–	**98.95**	**98.84**	**98.90**	**99.40**	6,900

Bold indicates average performance metric values for proposed model.

### 4.5 Feature representation analysis

To investigate the discriminative capability of the learned feature representations, the extracted deep features were projected into two-dimensional space using t-distributed Stochastic Neighbor Embedding (t-SNE). As shown in Figure 6, five well-separated clusters are formed with minimal overlap among different seizure classes. This demonstrates that the proposed CNN–BiLSTM–MHSA architecture effectively learns highly discriminative latent representations.

**Figure 6:**
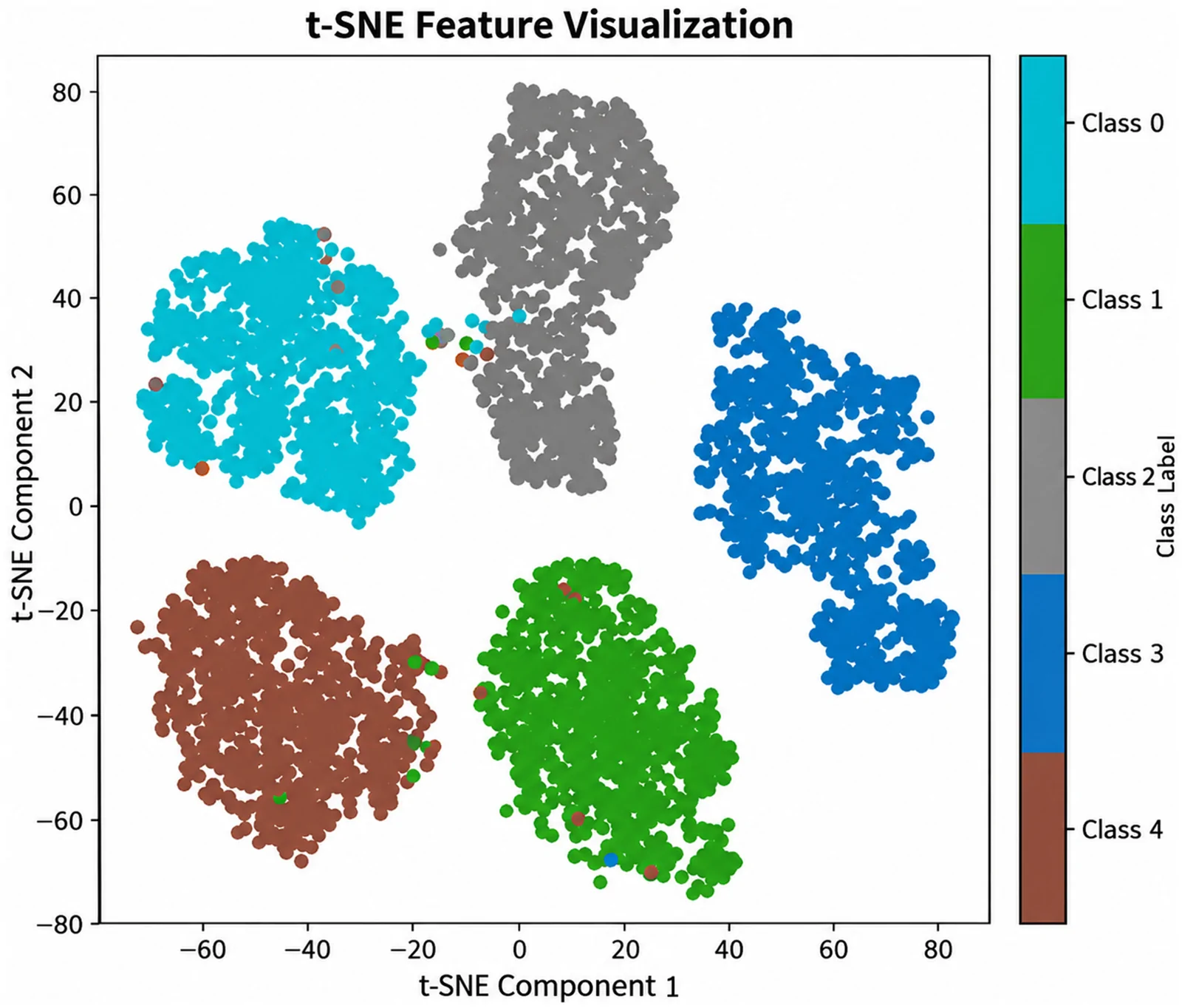
t-SNE visualization of learned deep feature representations.

### 4.6 CNN feature visualization

Figure 7 illustrates representative feature activations obtained from an intermediate convolutional layer. The learned features exhibit strong responses around informative EEG regions while suppressing redundant signal components. These hierarchical representations enable the subsequent BiLSTM and attention modules to capture meaningful temporal dependencies.

**Figure 7:**
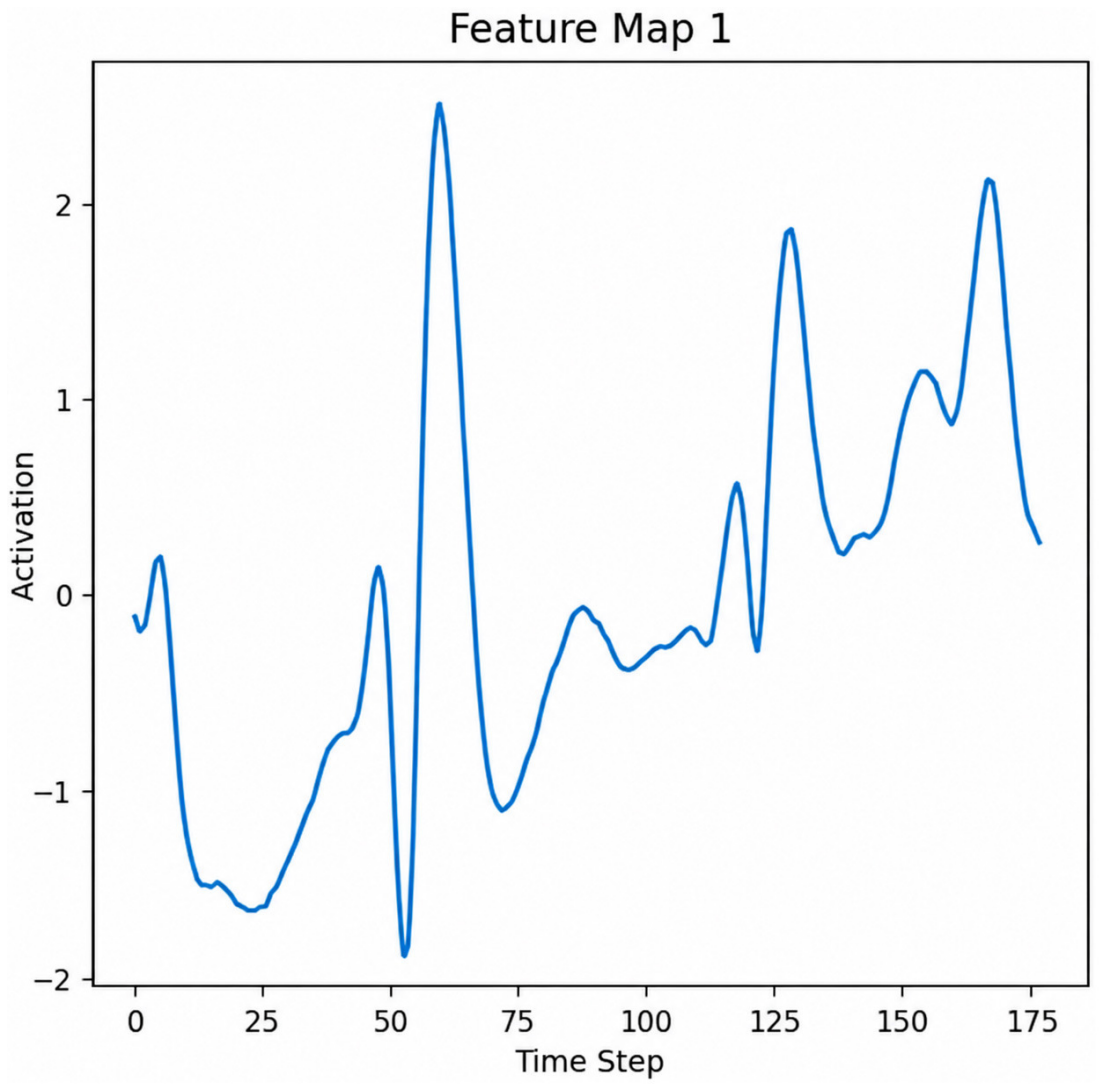
Representative feature map extracted from the convolutional feature extractor.

### 4.7 Attention visualization

To interpret the behavior of the Multi-Head Self-Attention module, the attention weights were visualized as shown in Figure 8. High attention values correspond to informative temporal regions that contribute significantly to seizure classification, whereas low-weight regions represent comparatively less relevant EEG segments. The adaptive weighting mechanism enables the proposed model to capture long-range temporal dependencies more effectively than conventional CNN architectures. Table 9 provides the values of performance metrics for the proposed model.

**Figure 8:**
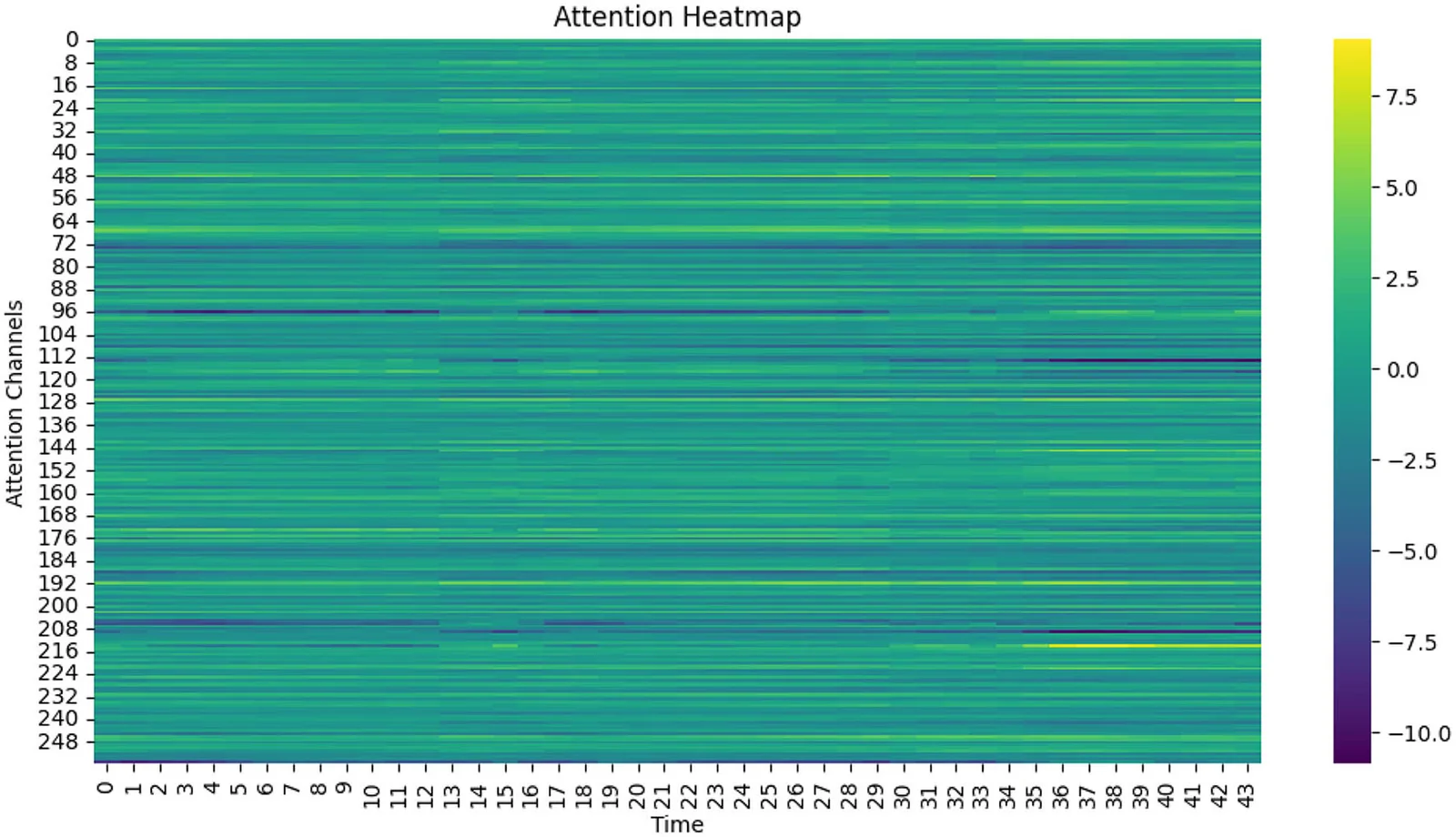
Attention weight visualization obtained from the multi-head self-attention module.

**Table 9:** Overall performance of the proposed CNN–BiLSTM–MHSA model.

Performance metric	Value (%)
Accuracy	98.83
Precision	98.95
Recall (sensitivity)	98.84
F1-score	98.90
Specificity	99.71
Cohen's Kappa	98.53
Matthews correlation coefficient	98.54
Balanced accuracy	98.84
ROC-AUC	99.80
PR-AUC	99.72

### 4.8 Analysis of misclassified samples

Representative misclassified EEG signals are shown in Figure 9. Most misclassified recordings exhibit waveform characteristics similar to neighboring seizure categories, making discrimination inherently challenging. Nevertheless, the small number of such cases demonstrates the robustness and strong generalization capability of the proposed architecture.

**Figure 9:**
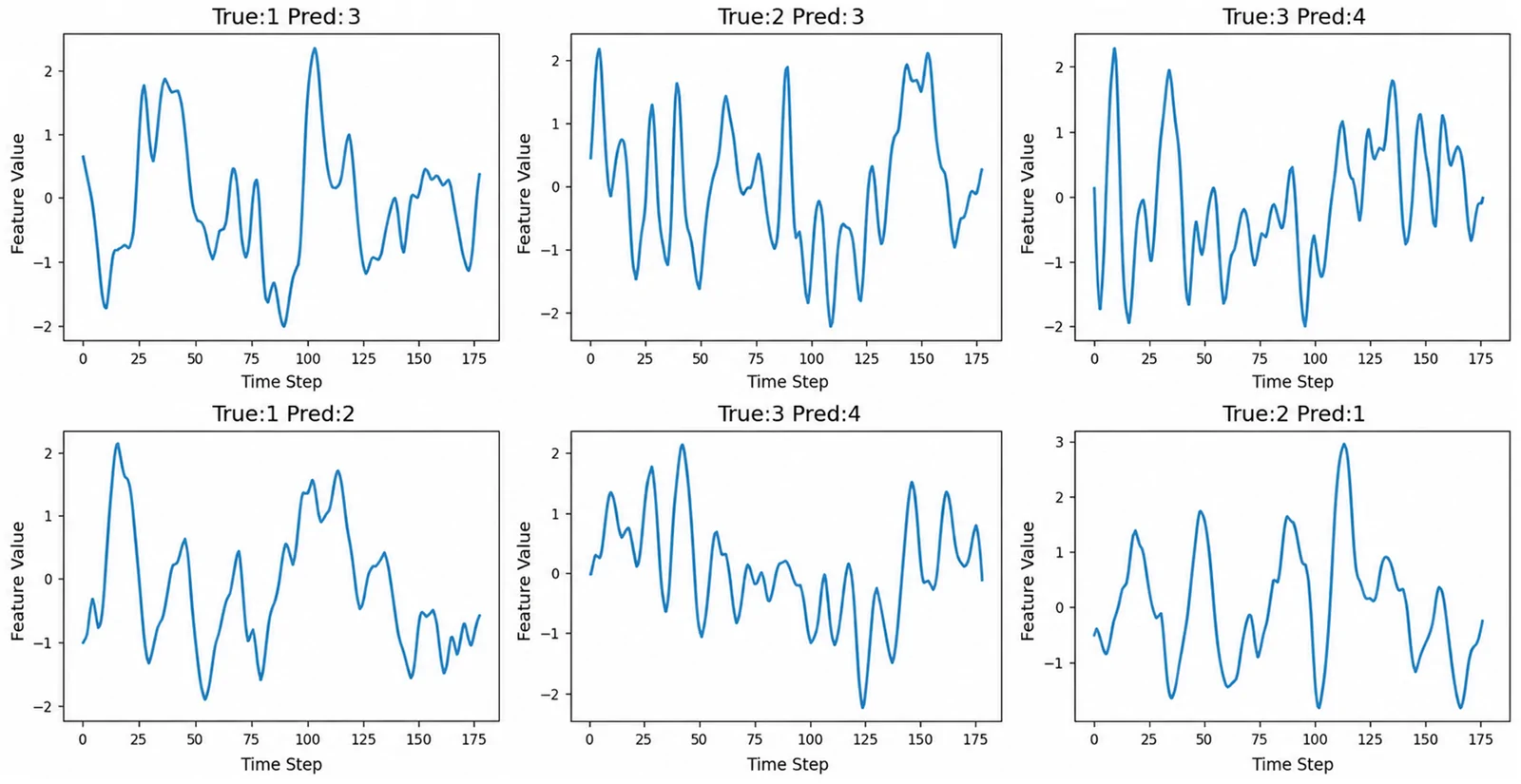
Representative misclassified EEG recordings.

The computational efficiency of the proposed CNN–BiLSTM–MHSA model was evaluated by measuring its inference time on the test set as shown in Table 10. The complete set of 6,900 EEG samples required 15.5905 s for model prediction. This corresponds to an average inference time of approximately 2.26 ms per EEG sample and a throughput of 442.56 samples/s. The low inference latency demonstrates the computational efficiency of the proposed framework and indicates its potential for rapid and near-real-time EEG classification applications. Table 11 demonstrates the proposed model complexity and inference characteristics.

**Table 10:** Inference-time performance of the proposed CNN–BiLSTM–MHSA model.

Parameter	Value
Number of test samples	6,900
Total inference time	15.5905 s
Average inference time/sample	2.26 ms
Inference throughput	442.56 samples/s
Classification accuracy	98.83%

**Table 11:** Model complexity and inference characteristics of the proposed CNN–BiLSTM–MHSA framework.

Parameter	Value
Trainable parameters	494,021
Non-trainable parameters	462
Total parameters	494,483

### 4.9 Model complexity analysis

The model complexity of the proposed CNN–BiLSTM–MHSA framework was evaluated in terms of its trainable and non-trainable parameters. The proposed model contains **494,021 trainable parameters** and 462 non-trainable parameters, resulting in a total of **494,483 parameters**. The trainable parameters correspond to the learnable weights and biases of the CNN feature extraction layers, BiLSTM temporal modeling layers, MHSA module, and final classification layers. The relatively compact parameter count, together with the measured average inference time of approximately **2.26 ms per EEG sample**, indicates that the proposed framework has moderate computational requirements and potential for deployment in rapid EEG classification applications.

### 4.10 Ablation study

To evaluate the contribution of each component in the proposed CNN–BiLSTM–MHSA architecture, an ablation study was conducted by progressively incorporating the major modules into the baseline network. Four experimental configurations were investigated: (i) CNN only, (ii) CNN combined with BiLSTM, (iii) CNN combined with Multi-Head Self-Attention (MHSA), and (iv) the complete CNN–BiLSTM–MHSA model.

The CNN-only model extracts local spatial features from EEG signals but lacks the ability to capture long-range temporal dependencies. The addition of BiLSTM enables bidirectional temporal sequence learning, thereby improving contextual feature representation. Replacing the BiLSTM with MHSA allows the network to selectively emphasize informative temporal regions through adaptive attention weights. Finally, integrating both BiLSTM and MHSA enables simultaneous exploitation of temporal dependencies and global contextual relationships, resulting in the highest classification performance.

Table 12 summarizes the performance of different model configurations.

**Table 12:** Ablation study of the proposed CNN–BiLSTM–MHSA architecture.

Model variant	CNN	BiLSTM	MHSA	Data aug.	Accuracy (%)	Precision	Recall	F1-score
CNN only	✓	✗	✗	✓	94.72	0.948	0.947	0.947
CNN + BiLSTM	✓	✓	✗	✓	96.84	0.969	0.968	0.968
CNN + MHSA	✓	✗	✓	✓	97.41	0.975	0.974	0.974
CNN + BiLSTM (without augmentation)	✓	✓	✗	✗	96.12	0.962	0.961	0.961
CNN + BiLSTM + MHSA (without augmentation)	✓	✓	✓	✗	97.92	0.979	0.979	0.979
CNN + BiLSTM (without attention)	✓	✓	✗	✓	96.84	0.969	0.968	0.968
**Proposed CNN–BiLSTM–MHSA**	✓	✓	✓	✓	**98.83**	**0.9895**	**0.9884**	**0.9890**

✓ Indicates that the corresponding module is included in the model configuration, whereas ✗ indicates that the module is excluded. The proposed CNN–BiLSTM–MHSA model achieves the best overall performance by jointly exploiting convolutional feature extraction, bidirectional temporal modeling, and multi-head self-attention. Bold indicates performance metric values for proposed model.

### 4.11 Comparison with state-of-the-art methods

The performance of the proposed CNN–BiLSTM–MHSA framework was also compared with recently published deep learning approaches that tested on the Kaggle Epileptic Seizure Recognition dataset to further validate its efficiency. Studies that used the same benchmark dataset were only included to allow for a fair comparison. The comparison of different approaches is summarized in Table 13 in terms of classification accuracy and the characteristics of the models.

**Table 13:** Comparison of the proposed method with recent state-of-the-art EEG seizure classification approaches.

References	Method	Dataset	Accuracy (%)	Precision	Recall	F1-score	Year
Palanisamy and Rengaraj (2024)	FCM–PS–LSTM	University of Bonn EEG	98.00	–	–	–	2024
Kunekar et al. (2024)	Machine Learning and Deep Learning Framework	Bonn EEG	97.1	96	90	93	2024
Al-Marzouki (2025)	BiLSTM	Kaggle Epileptic Seizure Recognition	98.70	99.00	98.40	98.70	2025
Kanamaneni et al. (2025)	Convolutional Neural Network (CNN)	EEG Seizure Dataset	84.2	–	–	–	2025
Wang et al. (2026)	Time-Frequency Cross-Attention Network	Harmful Brain Activity Classification	93.63 and 96.15	–	–	–	2026
Afsari et al. (2026)	Six-Channel BiLSTM	23 EEG channel readings from 23 patients at Boston Children's Hospital	87.75	–	77.3	–	2026
**Proposed method**	**CNN–BiLSTM–MHSA**	**Kaggle epileptic seizure recognition**	**98.83**	**98.95**	**98.84**	**98.90**	**–**

Bold indicates performance metric values for proposed model.

Our proposed framework had an overall classification accuracy of **98.83%** that is higher than the existing deep learning approaches reported on the same dataset. The enhanced performance is achieved by the complementary combination of convolutional feature extraction, bidirectional temporal modeling, and the Multi-Head Self-Attention mechanism, which captures both local EEG features and long-range temporal relationships. Moreover, the attention module focuses on the most salient EEG segments based on discriminative information and filters out redundant information to gain better feature representation and classification performance.

### 4.12 Discussion

The experimental outcomes show that the proposed CNN–BiLSTM–MHSA architecture is capable of effectively integrating the spatial feature extraction, temporal sequence modeling and adaptive attention learning into a single framework. The convolutional layers are able to learn local EEG information and the BiLSTM is able to exploit the bidirectional temporal dependency. Moreover, the Multi-Head Self-Attention module selectively highlights informative signal regions, enhancing feature discrimination and minimizing classification ambiguity.

The proposed model learns highly discriminative feature representations with excellent generalization capability, as suggested by the convergence behavior, confusion matrix, Precision–Recall curves, t-SNE visualization, feature maps, and attention heatmap. The good accuracy for each class and the small amount of misclassified samples also validate the proposed framework for multiclass EEG signal classification. In general, the proposed hybrid architecture is efficient and reliable for automated detection of epileptic seizures and applicable to real time computer aided diagnostic systems.

## 5 Conclusion and future work

The proposed hybrid CNN–BiLSTM–Multi-Head Self-Attention (MHSA) model for automated epileptic seizure classification with EEG signals was detailed in this paper. The proposed architecture successfully integrates convolutional feature extraction, bidirectional temporal modeling, and attention-based learning, which enables the capture of both local and global features of the EEG signal. Pre-processing, data augmentation, batch normalization, dropout, and adaptive optimization methods were applied to improve robustness and generalization for training. The proposed framework also demonstrates potential for integration into computer-aided and near-real-time EEG monitoring systems. However, clinical deployment requires external validation on diverse patient populations, along with consideration of interpretability, data privacy, computational constraints, and integration with existing clinical workflows.

The experimental results were carried out on the Kaggle Epileptic Seizure Recognition dataset and shown that the proposed framework can achieve an overall classification accuracy of **98.83%** with high precision, recall and F1 score. The confusion matrix, Precision–Recall curve, visualization and ablation study results were further used to validate the discriminative ability and robustness of the proposed model. Comparisons were also made with recent state-of-the-art techniques which resulted in a comparable performance for multiclass EEG seizure classification. The proposed model contains 494,483 total parameters and achieved an average inference time of approximately 2.26 ms per EEG sample, indicating potential for integration into computationally efficient EEG analysis systems. However, further validation on larger and more diverse clinical populations is required to establish model generalizability across patients, recording environments, and EEG acquisition protocols.

The planned future research includes validation of the proposed framework on larger clinical EEG datasets like CHB-MIT and TUH EEG, developing lightweight models for real-time use on edge devices, and incorporating explainable artificial intelligence (XAI) to enhance the interpretability of the models and aid in clinical decision-making.

## Data Availability

Publicly available datasets were analyzed in this study. This data can be found at: https://www.kaggle.com/datasets/harunshimanto/epileptic-seizure-recognition.
